# Intussusception after reconstruction following gastrectomy for gastric cancer

**DOI:** 10.1186/s12957-021-02456-3

**Published:** 2021-12-08

**Authors:** Feng Xia, Zhen Sun, Jian-Hong Wu, You Zou

**Affiliations:** 1 Hepatic Surgery Center, Tongji Hospital of Tongji Medical College of Huazhong University of Science and Technology, Wuhan, Hubei China; 2 Gastrointestinal Surgery Center, Tongji Hospital of Tongji Medical College of Huazhong University of Science and Technology, 1095, Jiefang Avenue, Wuhan, Hubei China

**Keywords:** Radical gastrectomy, Intussusception, Roux-en-Y, Billroth II, mechanism, diagnosis, treatment

## Abstract

**Background:**

Gastric cancer is the most prevalent tumor in Chinese men, and surgery is currently the most important treatment. Billroth II and Roux-en-Y are the anastomosis methods used for reconstruction after gastrectomy. Jejunal intussusception is a rare complication after gastric surgery.

**Main Body:**

Intussusception after gastric surgery occurs mostly at the gastrojejunostomy site for Billroth II reconstruction, and the Y-anastomosis site for Roux-en-Y reconstruction. Many studies have reported that postoperative intussusception appears at the anastomosis after bariatric surgery, while a few have reported intussusception at the anastomosis and its distal end after radical gastrectomy.

**Conclusion:**

A review was carried out to analyze intussusception after radical gastrectomy with roux-en-y anastomosis during the current situation. And the relevant mechanisms, diagnosis, treatment methods, etc. are described, hoping to provide better guidance for clinicians

## Introduction

Complications after gastric cancer surgery are often concerned by surgeons [1, 2]. Complications such as anastomotic leakage, infection, and postoperative bleeding occur after reconstruction. Intussusception of the bowel is defined as the telescoping of a proximal segment of the gastrointestinal tract within the lumen of the adjacent segment. Postoperative intussusception in adults is considered to be a relatively rare complication, accounting for less than 5% [3]. Although it only accounts for a small proportion, this has to attract our attention due to its relatively large pain and impact on patients and this review focuses on the rare complications of roux-en-y or BillrothII anastomosis and its distal intussusception after radical gastrectomy. At the same time, we explain the mechanism, risk factors, diagnosis, and treatment of postoperative intussusception in adults in detail, hoping to provide guidance for clinicians and reduce the adverse effects of intussusception on patients

The purpose of this review is to summarize the practical experience of this postoperative intussusception so that surgeons can make a better clinical judgment when managing patients who develop postoperative intussusception complications. In addition, we hope that our findings will guide future research on this topic.

### Intussusception and its related meanings

Intussusception is defined as the extension and retraction of a segment of the gastrointestinal tract in the lumen of an adjacent segment [4]. This condition is common in children and presents with typical crampy abdominal pain, bloody diarrhea, and a palpable tender mass. However, bowel intussusception in adults is considered a rare condition, accounting for 5% of all cases of intussusceptions and almost 1%-5% of bowel obstruction. Eight to twenty percent of cases are idiopathic, without a lead point lesion .

In children, it is usually primary and benign [5, 6]. In contrast, 90% of adult intussusception cases are secondary to certain pathological conditions, such as cancer, polyps, Merkel's diverticulum, colonic diverticula, strictures, or benign tumors, which are usually found intraoperatively, and less than 10% of adult intussusception occurs after gastric surgery [7–10].

### Mechanism of Intussusception

Intussusception is one of the causes of intestinal obstruction and is usually considered to be a pediatric disease. The main mechanism of intussusception in children is due to uncoordinated peristalsis and spasm of the intestinal canal. After an intestinal spasm, the spastic segment can be pulled by peristalsis into the immediate sphincter of the distal intestinal canal leading to intussusception, while spasm also occurs in the sphincter and forms a vicious cycle, which in turn becomes irreversible intussusception. But intussusception in adults is rare, especially after gastrectomy.

There is no clear mechanism in the previous literature that can explain the occurrence of postoperative intussusception, but the following three theories are currently popular [1]: Long input ends and excessive tissue inversion at the anastomotic site, postoperative adhesions, and stenosis can lead to impaired gastrointestinal function, including intestinal spasm, abnormal peristalsis [2]. Intestinal pacemaker interruption resulting in abnormal peristalsis. During the Roux-en-Y reconstruction, the distal jejunum is separated from the jejunal pacemaker during transection, resulting in decreased pacemaker potential distal to the anastomosis and activation of abnormal pacemakers in the distal jejunum. These ectopic pacemakers generate new pacing potentials that propagate more distally, resulting in delayed and stagnant emptying near the anastomosis [3]. Due to altered intestinal motility with dry and bruised bowel after prolonged and excessive manipulation, extensive preperitoneal separation, abdominal serum electrolyte levels, local hypoxia, anesthetics, and postoperative drug administration [11–15].

It is worth adding that several patients with intussusception in China had nasoenteric feeding tubes [16], and the intussusception was located at the anastomotic openings of the feeding tubes. Hu et al [17] reported seven cases of postoperative intussusception in adults caused by a nasoenteric feeding tube. The possible reason analyzed in this paper was that the feeding tube was pulled by manipulation, resulting in intestinal shrinkage and sleeving on the feeding tube, and the feeding tube was placed too deeply, resulting in the formation of stylet effect in the feeding tube in this segment of the intestinal canal. When there is a combined effect of the inducement of intestinal peristalsis disorder, the feeding tube serves as a support to the intestine, and it can also be a lead point for intussusception. In addition, very low temperatures of the nutrient solution easily promote intestinal spasms, and fast dripping speeds can induce intestinal peristalsis dysfunction. Notably, in bariatric surgery, intussusception after the Roux-en-Y gastric bypass procedure increases the risk of stretching and intussusception due to rapid weight loss in addition to the above reasons [18–22].

### Risk factors for postoperative intussusception

Some studies suggest that patients who undergo Roux-en-Y and BII anastomosis during surgery are prone to small bowel intussusception. Narasimhan et al. on the other hand concluded that Roux-en-Y anastomosis is more likely to develop postoperative small bowel intussusception than BI and BII anastomosis.

Epidemiological studies [23] have shown that young, female, obese patients are prone to postoperative intussusception. This may be related to different surgical methods and different study populations. In general, relaxation of the mesentery will weaken its supporting and fixation effect on the intestine, which increases the range of motion of the intestine and makes it prone to small bowel intussusception. The mesentery is usually relaxed and thin in patients with a low BMI. Mesenteric relaxation also commonly occurs in patients with a large reduction in body mass within a short period. The existing reports of postoperative small bowel intussusception are mainly from Europe and the United States. Young obese women who undergo bariatric surgery often experience a significant decrease in body weight after surgery [24], so it is concluded that young, female, and obese patients are prone to postoperative intussusception. In China, gastrectomy and gastrointestinal anastomosis are mostly used in digestive system tumor surgery, and patients often have a low preoperative BMI, so they rarely experience a significant decrease in postoperative body mass. In addition, patients with high BMI have relatively thick mesentery and fixed support for the bowel, so they are not prone to postoperative small bowel intussusception [25].

At the same time, according to a study in a domestic hospital in China [25], intussusception on the stapler during surgery is also a risk factor for the development of postoperative intussusception.

### Diagnosis of postoperative intussusception

Small bowel intussusception after gastric cancer surgery is a rare complication, postoperative inflammatory adhesions are mostly considered when intestinal obstruction occurs after surgery and its presentation is often atypical. Patients often do not have classical abdominal pain, nausea, bloody stools, abdominal symptoms, and other symptoms. Most patients only present with persistent abdominal pain and nausea. In laboratory tests, inflammatory indicators and infectious indicators are often negative, so early diagnosis of postoperative small bowel intussusception is difficult.

Therefore, it is often misdiagnosed as postoperative "anastomotic edema" and "adhesive intestinal obstruction". Se-Youl Lee et al [11] reported that a 77-year-old woman complained of abdominal pain on the fifth day after radical total gastrectomy, and swallowing imaging studies with water-soluble contrast agents indicated intestinal obstruction. Akira Yoneda et al [26] reported that an 80-year-old woman was transferred to the hospital due to abdominal pain and nausea. She underwent total gastrectomy and reconstruction 14 days earlier and was treated for gastric cancer with a modified BillrothII method. An active mass was palpated in the left lower quadrant, there was tenderness in the upper abdomen, and intussusception was found by exploratory laparotomy. Postoperative intussusception mostly occurs when liquid diet has just been resumed after the operation. At present, the application of auxiliary examination is timely, and intestinal necrosis does not occur. Hence, bloody stools are rare in clinical practice. Therefore, when the symptoms of high intestinal obstruction occur after Roux-en-Y anastomosis after gastrectomy, the possibility of intussusception should be considered. Typical intussusception on abdominal CT shows "kidney sign", "double tube sign" or "target sign", and sometimes pneumatosis intestinalis is seen. Typical intussusception images by B-mode ultrasound show "concentric circle sign", "ring target sign", and "sleeve sign" and Barium meal examination shows cup-shaped filling defect at the site of obstruction, bifurcation sign, line-like sign, parallel double line sign or efferent loop truncation [3, 27–30]

### Manangement of postoperative intussusception

Several studies [13, 31, 32] indicate that postoperative intussusception should be treated with early surgical intervention, early multi-energy manual reduction, or resection and anastomosis when a blood circulation disorder or even necrosis occurs in the intussuscepted bowel (generally, the intussuscepted bowel has no blood circulation disorder). Laparoscopic surgery for simple intussusception has minimally invasive advantages, but intussusception caused by complex causes should be treated according to the situation. It is still controversial whether intestinal resection should be performed after reduction to prevent recurrence, and it is believed that reduction alone? can be performed if there is no intestinal blood circulation disorder, but some scholars prefer intestinal resection [33–35].

At present, the main methods to treat postoperative intussusception are digestive endoscopic treatment and surgical treatment

#### Digestive endoscopy

Reduction using digestive endoscopy has been reported in some previous case reports. The basic procedure includes the reduction of intussusception, as well as the placement of a jejunal feeding tube deep into the input loop [14, 26, 36]. The adhesion between the feeding tube and the intestinal wall can play a fixation role, and then the nutrition through the feeding tube can give the digestive tract enough time to restore normal peristaltic function. However, some reports recommend this operation as a temporary measure before surgical correction [37]. If there is a suspicion of gastrointestinal perforation, anastomotic leakage, and the patient is still in the perioperative period, the risk is high, and the recurrence rate after endoscopic reduction is high, it is therefore not currently used as the first choice of treatment in these patients [38–40].

#### Surgery

The surgical approach to postoperative intussusception, including reduction, resection, removal of the limb, and revision of the anastomosis, depends on intraoperative findings [27, 29, 41–45]. Fixation of the reduced jejunum to adjacent tissues, such as the mesocolon, colon, or stomach, may be considered to prevent recurrence [14, 46]. Seventy percent of chronic small bowel intussusceptions have the possibility of conversion to acute intussusception, and chronic intussusception can present with recurrent attacks without treatment, so once diagnosed, therapeutic measures should be taken as soon as possible. In acute postoperative small bowel intussusception, the risk of surgery, and mortality are closely related to whether surgical treatment can be performed in time. Patients who undergo surgery within 48 hours after the onset of symptoms have a mortality rate of less than 10%, while those who undergo surgery more than 48 hours have a mortality rate as high as 50% [14]. Therefore, if the symptoms are not relieved by strict medical treatment for 48 hours, prompt surgery should be performed to avoid further delaying treatment and prevent adverse consequences. As mentioned above, the surgical treatment of postoperative intussusception should be decided according to the patient's condition. The timing of surgery should be carefully selected. For patients with chronic postoperative small bowel intussusception, or early acute postoperative small bowel intussusception, who have not yet experienced intestinal ischemia or necrosis, the surgical treatment is simple surgical reduction, and the action should be gentle and slowly advanced, so as not to damage the intestinal canal or even possible tumors [47, 48].

If intestinal constriction is discovered during surgery and it is judged that it will be difficult to restore the activity of the intestinal canal, or the intussuscepted intestinal canal cannot be reduced, the necrotic intestinal canal or intussuscepted intestinal canal is removed [49–51]

Raj Gopal et al [52] reported a case of retrograde jejunal intussusception discovered? by CT after Billroth II anastomosis after gastrectomy. Emergency exploratory laparotomy, found retrograde intussusception of the distal jejunum, gangrenous intussusception at the jejunojejunostomy and gastrojejunostomy. This was also the first report of retrograde intussusception across two anastomoses, after which the surgeon resected the local intestinal segment and the intestinal continuity was restored by two jejunojejunostomy

In a single-center study involving 15,000 patients by Steven C. Simper M.D [53], 35% of patients experienced bowel narrowing and underwent bowel resection. For patients requiring resection of the bowel distant from the anastomosis, continuity is re-established by jejunojejunal end-to-end anastomosis after resection [54–57], and the intussuscepted bowel should be carefully examined during surgery for new tumors, adhesions, lymphoproliferative Ascaris lumbricoides, lymphadenopathy, and other factors that may affect intussusception [58].

The author's team had a patient who underwent total gastrectomy due to gastric tumor and was reconstructed by the Roux-en-Y method. The patient developed vomiting, abdominal pain, and abdominal distension on the third day after the operation. Empirical abdominal CT was performed. The results showed intussusception. After diagnosis, gastroscopic recovery was attempted. The symptoms were relieved after gastroscopy. However, intussusception recurred on the third day after the operation. Finally, manual reduction was performed and the patient's prognosis was good.

#### Other treatment modalities

In chronic or postoperative intussusception, medical treatment can be attempted, including intravenous fluids, nasogastric tube aspiration, and endoscopic or barium procedures. In the meantime, it is well documented that glucagon-induced hypotension may promote reduction [14, 59], but this treatment modality is not advocated nowadays because the possibility of recurrence is still quite high.

In China, Li et al. [60] reported a case of distal subtotal gastrectomy due to cancerous gastric antrum. On the third day after Billroth jejunostomy, CT showed efferent loop obstruction. The patient was given correction of hypoproteinemia, and surgical treatment was planned. The patient refused invasive treatment and was monitored for changes in the condition. The nasoenteric tube was removed on the tenth day after the operation, and the patient had no discomfort in eating. CT re-examination of did not show intussusception. In this case, conservative treatment was successful and has not been reported at home and abroad before

After many years of development in the treatment of postoperative intussusception in adults, most of the published literature reports that surgical treatment should be performed [28, 61–66]. The summary of the treatment methods for postoperative intussusception in adults is as follows [1]: conservative treatment can be performed first for small bowel intussusception, followed by surgical reduction or resection when ineffective [2] for patients with short onset time, gastroscopic dilatation, inflation therapy, and pushing ring can be tried within 48 hours of onset [3] emergency surgical resection should be performed if there is significant intestinal inflammation, ischemic necrosis, and CT indicates tumor or other induction points [4] other conditions can be surgically manipulated by? reduction [28–30, 67]

### Prevention of postoperative intussusception

For patients undergoing gastrectomy, it should be noted during the operation that [1] The jejunal feeding tube should not be inserted too close to the anastomosis [2]; the tension should be balanced during jejunal side-to-side anastomosis, and the intestinal canal should not be suspended at an angle [3]; the intraoperative maneuvers should be gentle to minimize the stimulation to the intestinal canal, and the small intestine itself should be carefully examined for pathological changes [4]; when inserting the nasoenteric feeding tube, the intestinal canal should not be forcefully pushed and pulled to send the feeding tube to the distal end, and the other professionals in the operating room should inject normal saline into the feeding tube to dilate and insert the small intestine at the same time, the surgeon cooperates to straighten the feeding tube, prevent bending, and also avoid intestinal shrinkage and overlap [5]; the anastomosis should not be too long, 10 cm-15 cm can be used [68]. The jejunal feeding tube should be properly fixed in the perioperative period; the balloon of the jejunal feeding tube should not be overfilled to reduce the irritation to the small intestinal wall during catheterization. After reconstruction, the patient should get out of bed as early as possible, or they should be in a semi-decubitus position to prevent intra-abdominal adhesions. At the same time, the starting time and speed of feeding through the jejunal feeding tube should be adjusted, which should not be premature. Feeding should be started when the patient has spontaneous peristalsis in the gastrointestinal tract. The infusion rate of nutrition should be coordinated with the degree of recovery of gastrointestinal function; the jejunal feeding tube should have a sufficient number of side holes, as well as the stimulation of dispersed food to the small intestinal wall. After gastrojejunal reconstruction, the patient should eat small meals and less irritating food [17].

## Conclusion

Intussusception after gastrectomy is a rare but challenging condition for surgeons. Postgastrectomy intussusception is usually subacute, and it presents without the specific clinical manifestations associated with intussusception in children, hence, the diagnosis of postgastrectomy intussusception is usually delayed or missed. Abdominal CT is considered to be the most sensitive examination for the diagnosis of intussusception and it can determine the location of intussusception as well as the presence or absence of induction points. The possibility of small bowel intussusception should be considered when patients have persistent abdominal pain after gastrectomy. Early identification by abdominal CT examination can help achieve timely treatment ad avoid missed diagnoses. Surgical intervention is mandatory because mortality rises dramatically after more than 48 hours of intussusception following gastrectomy. Surgical treatment is usually manual reduction. If necrosis of the bowel segment is found, the necrotic bowel segment can be removed locally.

## Data Availability

Data sharing is not applicable in this article as no datasets were generated or analyzed during the current study.

## Abbreviations

MRI: Magnetic resonance imaging
CT: Computed tomography
GI: gastrointestinal
BMI: Body Mass Index
